# Elemental or contextual? It depends: individual difference in the hippocampal dependence of associative learning for a simple sensory stimulus

**DOI:** 10.3389/fnbeh.2014.00217

**Published:** 2014-06-16

**Authors:** Kyung J. Lee, Seong-Beom Park, Inah Lee

**Affiliations:** Department of Brain and Cognitive Sciences, Seoul National UniversitySeoul, South Korea

**Keywords:** hippocampus, contextual memory, associative learning, muscimol, rodent, conditioning

## Abstract

Learning theories categorize learning systems into elemental and contextual systems, the former being processed by non-hippocampal regions and the latter being processed in the hippocampus. A set of complex stimuli such as a visual background is often considered a contextual stimulus and simple sensory stimuli such as pure tone and light are considered elemental stimuli. However, this elemental-contextual categorization scheme has only been tested in limited behavioral paradigms and it is largely unknown whether it can be generalized across different learning situations. By requiring rats to respond differently to a common object in association with various types of sensory cues including contextual and elemental stimuli, we tested whether different types of elemental and contextual sensory stimuli depended on the hippocampus to different degrees. In most rats, a surrounding visual background and a tactile stimulus served as contextual (hippocampal dependent) and elemental (non-hippocampal dependent) stimuli, respectively. However, simple tone and light stimuli frequently used as elemental cues in traditional experiments required the hippocampus to varying degrees among rats. Specifically, one group of rats showed a normal contextual bias when both contextual and elemental cues were present. These rats effectively switched to using elemental cues when the hippocampus was inactivated. The other group showed a strong contextual bias (and hippocampal dependence) because these rats were not able to use elemental cues when the hippocampus was unavailable. It is possible that the latter group of rats might have interpreted the elemental cues (light and tone) as background stimuli and depended more on the hippocampus in associating the cues with choice responses. Although exact mechanisms underlying these individual variances are unclear, our findings recommend a caution for adopting a simple sensory stimulus as a non-hippocampal sensory cue only based on the literature.

## Introduction

It has long been suggested that there are two systems for associating external stimuli with reinforcement (Rudy and Wagner, 1976; Sutherland and Rudy, 1989; Fanselow, 1999): one system, the elemental learning system, is responsible for associating an elemental stimulus such as a single tone or light with a reinforcer (Estes, 1950; Rescorla and Wagner, 1972) and the other, the configural or contextual system, is responsible for associating a more complex stimulus, such as a visual scene in the background, with a reinforcer (Rudy and Sutherland, 1989; Fanselow, 1999). Whether the two learning systems always compete with each other or function in parallel is still debatable although it has been suggested that the existence of a contextual stimulus prevents an elemental stimulus from controlling behavior (Fanselow, 1999).

The hippocampus is considered to be a part of the contextual learning system and almost exclusively involved in dealing with complex stimuli that are composed of a myriad of elemental subcomponents (Fanselow, 1999). The competition between elemental and contextual stimuli has been tested typically in a fear-conditioning paradigm in which an animal is trained to freeze in response to either an elemental stimulus (e.g., tone or light) or contextual stimulus (e.g., room cues mixed with odors). Many studies have demonstrated that rats with hippocampal lesions exhibit impairments for associating contextual stimuli with freezing, but no impairments when associating elemental stimuli with freezing (Kim and Fanselow, 1992; Phillips and LeDoux, 1992, 1994). The fear-conditioning literature appears to have reached a consensus that simple tones and lights are elemental stimuli, and thus non-hippocampal dependent, whereas complex stimuli such as room cues in the animal's background are considered contextual stimuli and thus hippocampal dependent. However, one needs to be cautious when unequivocally labeling a certain type of stimulus as contextual or elemental (or hippocampal or non-hippocampal) on the basis of the results from a particular behavioral paradigm such as the fear-conditioning task. For example, classical eyeblink conditioning paradigms reliably recruit the hippocampus in the absence of the contextual requirement especially when a temporal gap (i.e., trace) is inserted between a conditional stimulus (CS) and an unconditional stimulus (US; Gruart et al., 2006; Nokia and Wikgren, 2014). Studies based on the configural learning theory (Rudy and Sutherland, 1989) also examined whether hippocampal-lesioned rats could associate elemental stimuli (e.g., light and tone) with a behavior (mostly lever pressing) and their configural form (e.g., light+tone) with the inhibition of that behavior, but this line of studies produced mixed results (Sutherland et al., 1989; Whishaw and Tomie, 1991; Gallagher and Holland, 1992; Davidson et al., 1993).

Considering that the hippocampus has long been one of the major brain regions investigated for the neural mechanisms of memory, it is very important to clearly define the type of external stimuli that require the hippocampus for learning. In the current study, we reexamined this issue using memory tasks in which rats were required to associate various types of so-called elemental and contextual stimuli with distinct behaviors.

## Materials and methods

### Subjects

Sixteen rats (Long-Evan, male, 280–380 g) were used in this study. All animals were maintained on a 12-h light/dark cycle. Food was restricted to maintain rats at 80% of their free-feeding weight during behavioral testing. All protocols were approved by the Institutional Animal Care and Use Committee of the Seoul National University.

### Apparatus

Detailed descriptions of the behavioral apparatus were provided in previous reports (Kim et al., 2012; Lee and Shin, 2012). Briefly, a rectangular platform (14.5 × 29 cm) surrounded by an array of liquid crystal display (LCD) monitors was used for behavioral tasks throughout the study. A jar filled with a mixture of play sand and Froot-Loops (Kellogg's) cereal powder was placed in a fixed location on the platform, covering a food well. A visual context was provided by displaying a visual scene (a zebra stripe or a pebbles pattern) on the LCD screens. A small light bulb (1-cm diameter, 1.5 V, 0.3 A) was attached to a transparent wall at the edge of the platform to provide a simple light stimulus. A pair of computer speakers was placed underneath the platform to provide a simple tone stimulus. A trial started when an experimenter opened a start box attached to the platform, allowing the rat to enter the platform.

### Behavioral paradigm

**Figure 1:**
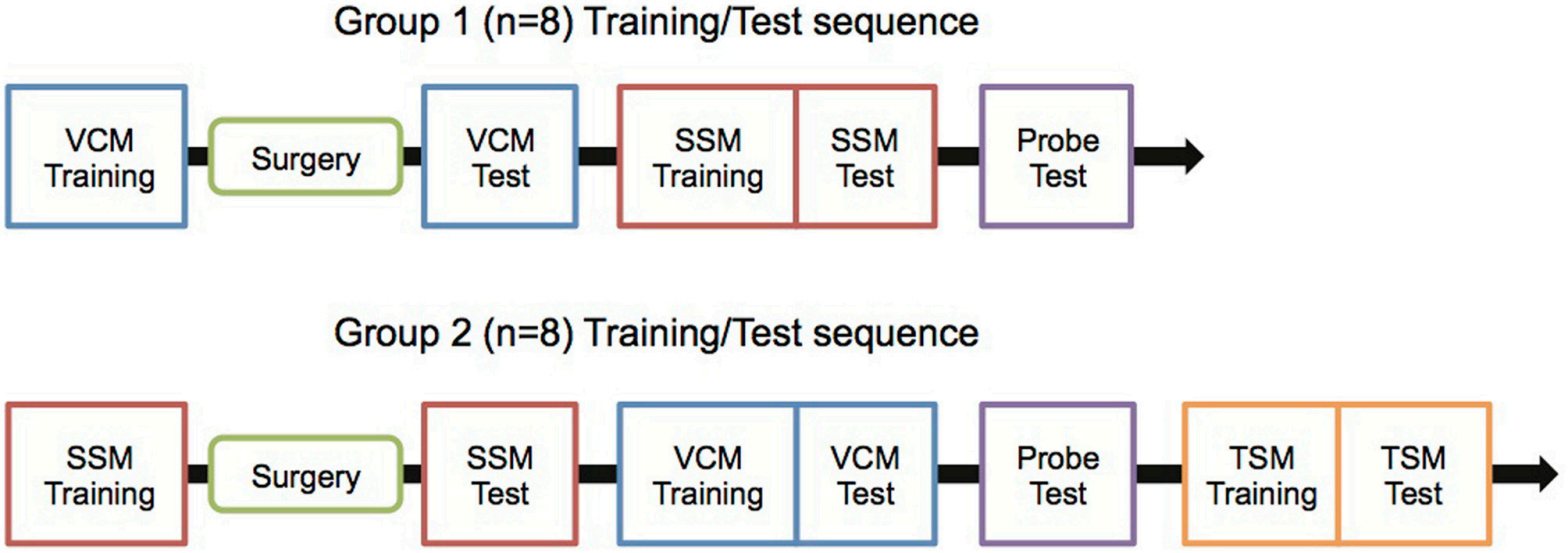
**Experimental schedules**. Rats were trained with the experimental schedule either for Group 1 or Group 2 to control the possibility that the sequence of behavioral training (between VCM and SSM tasks) might affect the testing results. VCM, Visual Contextual Memory; SSM, Simple Stimulus Memory; TSM, Tactile Stimulus Memory.

Overall experimental schedules are illustrated in Figure 1. In the current study, all rats were trained in a certain behavioral task (see below) before being surgically implanted with hippocampal cannulae. Then, the rats were tested in that behavioral paradigm only. Afterwards, the rats were trained in another behavioral task and were tested once they reached performance criterion. Specifically, Group 1 (*n* = 8) was trained in a visual context memory (VCM) task, surgically implanted with bilateral cannulae in the hippocampus, and then was tested in the VCM task after recovery. The rats in group 1 were then trained and tested in a single stimulus memory (SSM) task, and a probe task was carried out after the SSM task. Group 2 (*n* = 8) was first trained in the SSM task, implanted with cannulae, and tested in the SSM task. Then, the rats in Group 2 were trained and tested in the VCM task before they were subjected to the probe task. Group 2, but not Group 1, was also trained and tested in a tactile stimulus memory (TSM) task afterwards.

### Pre-surgical behavioral training

#### VCM task

**Figure 2:**
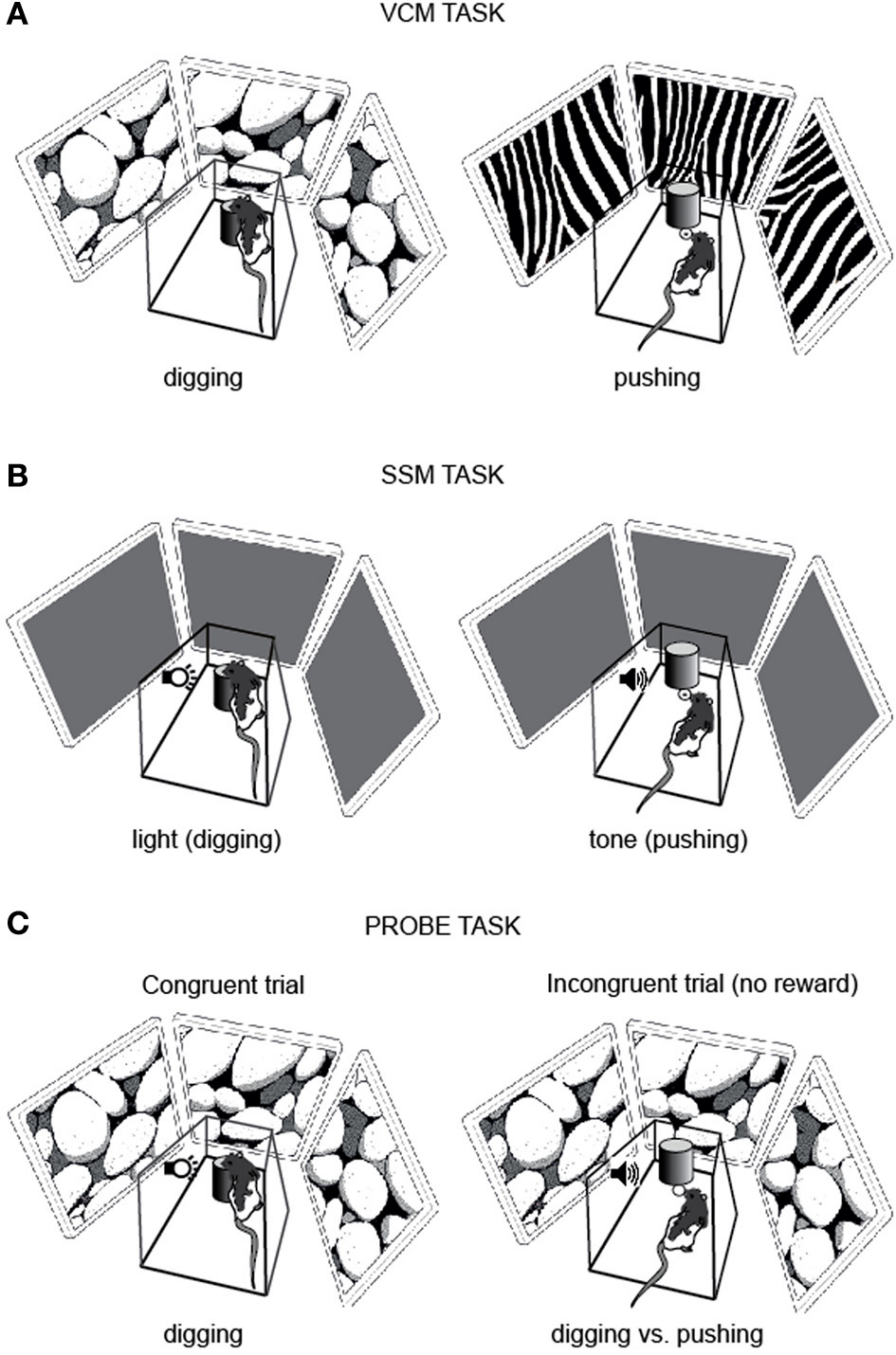
**Behavioral paradigms. (A)** VCM task. To obtain a reward, the rat was required to dig in the sand in the jar (digging, *left*) or push the jar (pushing, *right*) in association with the visual scene (pebbles or zebra pattern) displayed on the LCD panels surrounding the platform. **(B)** SSM task. The rat was required to dig in the sand in the jar (digging, *left*) or push the jar itself (pushing, *right*) in association with the simple sensory cue (light or tone). **(C)** Probe task. A visual context and a simple sensory cue were simultaneously presented. In congruent trials (*left*) the visual context (pebbles in this example) and the simple sensory cue (e.g., light) were associated with the same response (e.g., digging) and a reward was given to the rat regardless of the choice response. In incongruent trials *(right*) the visual context (pebbles in this example) and the simple sensory cue (e.g., tone) were associated with the opposite responses (e.g., digging for pebbles and pushing for tone) and no reward was given to the rat for either response.

Rats were trained to dig in a sand jar to retrieve a piece of Froot Loops (Kellogg's) cereal when one visual scene (e.g., pebbles) was displayed on the LCD screens and to push the same jar to gain access to the food well beneath the jar when the other visual scene (e.g., zebra stripes) was displayed (Figure 2A). Therefore, in the VCM task, the rat was required to use the visual scene information in its background (i.e., visual context) to remove ambiguity in response associated with the object and make a proper behavioral choice.

The visual scene stimulus was displayed as soon as the start box door was opened. Four rats learned to associate the digging behavior with the zebra pattern and the pushing behavior with the pebbles pattern, and the other four learned the opposite stimulus-response contingency. Response latency was measured with a stopwatch from the platform entry to either touching the sand with a front paw (considered as digging) or pushing the jar. Once the rat made a wrong response by touching the sand when pushing the jar was appropriate or by pushing the jar when digging was the correct response, the experimenter stopped the animal with a small plastic panel (not allowing the rat to obtain food or to correct its response) and gently guided the rat to the start box. Thirty-two trials were performed in a session and the two visual scenes appeared with equal probabilities. Rats were trained to criterion (≥75% correct performance for both pushing and digging behaviors for 2 consecutive days) before receiving surgery.

#### SSM task

The task and training procedures for the SSM task (Figure 2B) were exactly the same as described above for the VCM task, except that the LCD screens remained black and did not show any visual pattern. The visual scene stimuli were replaced with simple sensory stimuli: a light and a tone (sine wave, 800 Hz). The light or tone stimulus was turned on immediately after the start box door was opened and was terminated once the rat made a response.

### Surgery

Rats were implanted with bilateral guide cannulae (26G, coupled with 32G stylets) in the dorsal hippocampus in a stereotaxic frame, following the procedures previously described by Kim et al. (2012). The cannulae targeted 3.9 mm anterior to bregma, 2.6 mm lateral to midline, and 3.0 mm ventral from the skull surface. The cannulae were fixed with dental cement and anchoring screws on the skull. Rats were allowed to recover for 7 days before post-surgical testing.

### Post-surgical behavioral testing with intracranial drug injection

After recovery from surgery, rats were retrained to criterion (75% correct responses) in the same task in which they had been trained before surgery (i.e., VCM for Group 1 and SSM for Group 2). Once they reached performance criterion, they were tested 20 m after injection of phosphate-buffered saline (0.5 μL, 10 μL/h rate) into the hippocampus. On the following day, they were tested 20 m after being injected with muscimol (0.5 μg/0.5 μL), a GABA-A receptor agonist, into the hippocampus for the temporary inactivation of the hippocampus.

After post-surgical testing, rats were trained in the other task (i.e., SSM task for Group 1 and VCM task for Group 2). Once trained to criterion (75% correct responses), rats were tested with the same drug-injection schedules (saline followed by muscimol). One of the rats in Group 2 died accidentally before being tested in the VCM task, and the data were analyzed only in the SSM task for that rat.

#### Probe task

After rats were tested in both the VCM and SSM tasks, they performed a probe task for 3 days for examining the competition between associative memories for visual contexts and simple sensory stimuli (Figure 1). On day 1, the rat performed 50 trials in which the visual scene and simple sensory stimuli that required the same response (e.g., digging) were simultaneously presented and associated with a reward (Congruent trial; Figure 2C). On day 2, saline was injected into the hippocampus and, afterwards, the rat performed 50 congruent trials and 10 incongruent trials. In the incongruent trials, the visual scene and simple sensory stimuli that required opposite responses (digging and pushing) were simultaneously presented. No reward was provided for the incongruent trials and we analyzed only the first incongruent trial to exclude any learning effect. The congruent and incongruent trials were intermixed in a random sequence. On day 3, the same task (50 congruent trials and 10 incongruent trials) was performed with muscimol injection.

#### TSM task

After being tested for 3 days in the probe task, rats in Group 2 (*n* = 7) were further trained in the TSM task (Figure 7A). The responses required in the TSM task were the same (digging and pushing), but the cueing stimulus was a floor insert (13 × 10 cm) covered with either sandpaper or wire mesh. The tactile stimulus was laid on the floor of the platform before a trial started. Neither a visual context nor a tone or light stimulus was provided in the TSM task. Once rats were trained to criterion (≥75% correct performance for both tactile stimuli for 2 consecutive days), they were tested after saline and muscimol injections as in the SSM and VCM tasks.

### Histology

**Figure 3:**
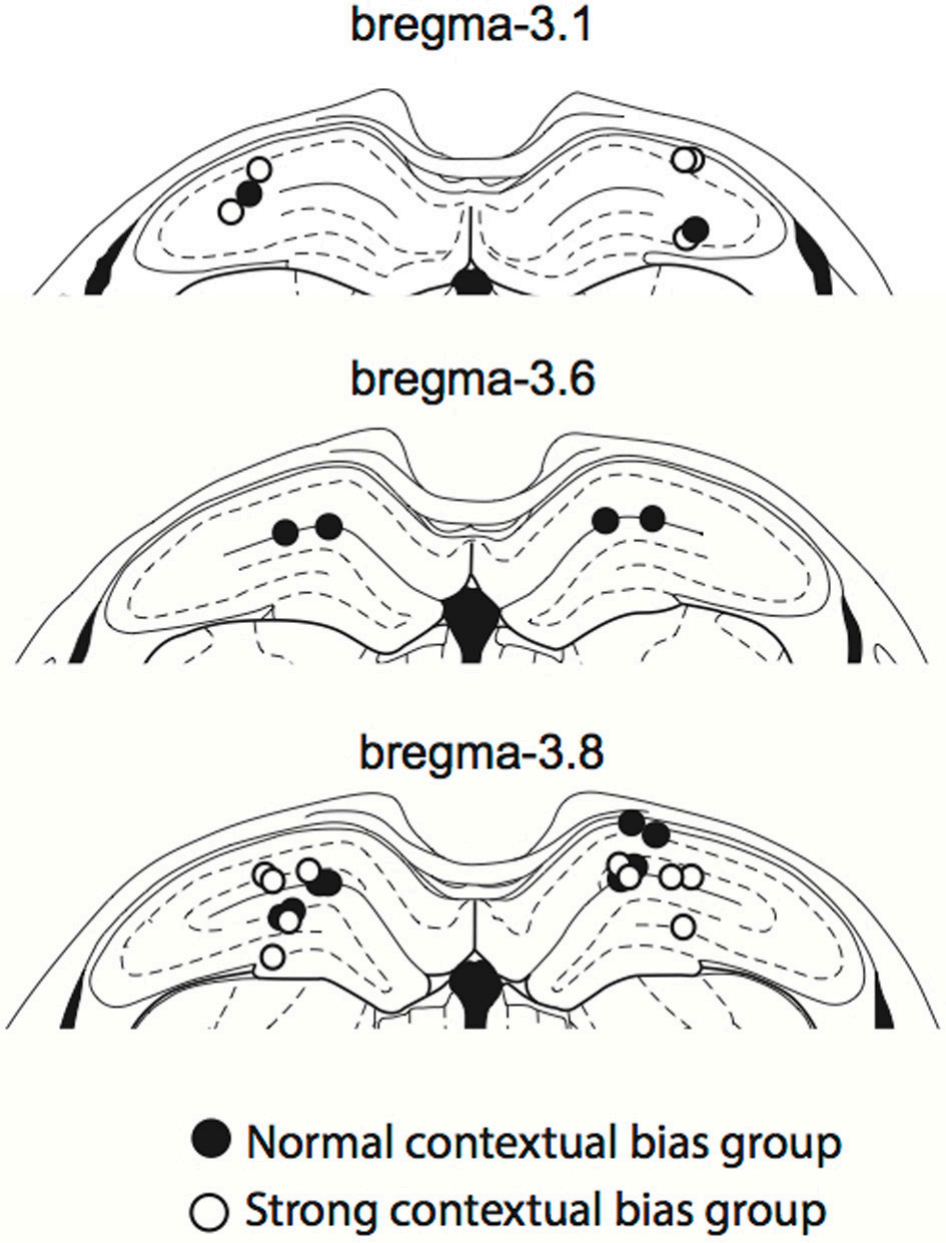
**Cannula positions**. The cannula tip positions for all rats used in the study. All cannula tips were located within the dorsal hippocampus, ranging from 3.1 to 3.8 mm anterior to bregma. Colors denote normal (filled circles) and strong (open circles) context groups (see the main text for details).

After all behavioral experiments were completed, rats were killed with CO_2_ inhalation and perfused transcardially with 0.9% saline and 4% formaldehyde solution. The brain was extracted and cryoprotected in 30% sucrose-formalin solution before being sectioned in a sliding microtome (40-μm thickness). The cut sections were Nissl-stained with thionin and the cannula tip positions were verified with a light microscope. Tips of all cannulae were within the dorsal hippocampus (Figure 3).

### Data analysis

Two-Way mixed ANOVAs were used to compare performance and response latency across drug condition (within-subjects factor; saline, muscimol) and task order (between-subjects factor; VCM-SSM, SSM-VCM).

## Results

### Both contextual and elemental stimuli required the hippocampus

**Figure 4:**
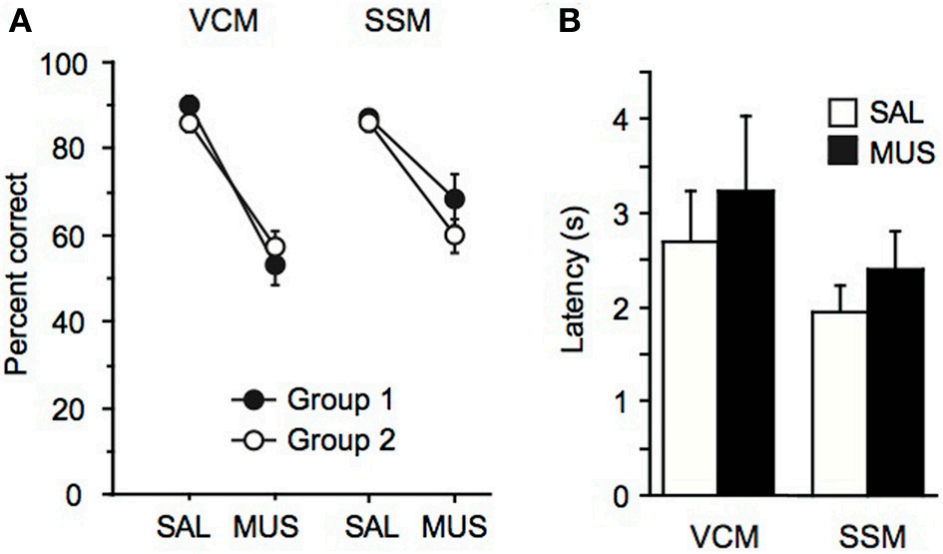
**Performance in the VCM task and the SSM task. (A)** *Left*: Performance (percent correct trials) in the VCM task when saline (SAL) or muscimol (MUS) was injected into the dorsal hippocampus in rats trained and tested in the VCM task before being trained and tested in the SSM task (Group 1; filled circle) and in rats trained and tested in the SSM task before being trained and tested in the VCM task (Group 2; open circle). *Right*: Performance in the SSM task with the same presentation scheme as in the VCM task. **(B)** Response latency in the VCM task and in the SSM task, when SAL or MUS was injected into the dorsal hippocampus. All graphs show mean ± s.e.m.

When injected with saline, rats showed normal performance (approximately 90% correct response level) in the VCM task. However, muscimol injections on the next day produced severe deficits in performance (Figure 4A). There was a significant effect of drug condition [*F*_(1, 13)_ = 124.2, *p* < 0.0001], but no significant effect of group (i.e., group 1 vs. group 2) and no interaction effect between group and drug condition (*p* > 0.1). There was no significant effect of drug condition on response latency in the VCM task [*F*_(1, 14)_ = 0.5, *p* = 0.5; Figure 4B].

Similarly, rats were not affected by saline injections in the SSM task (approximately 90% correct response level), but, on the next day, the performance of the same animals dropped markedly when muscimol was injected 20 m before testing (Figure 4A). There was a significant effect of drug condition [*F*_(1, 14)_ = 30.3, *p* < 0.0001] but there were no significant effect of group and no interaction between group and drug condition (*p* > 0.1; Figure 4A). The average performance under muscimol in the SSM task was significantly higher than the MUS performance in the VCM task [*t*_(14)_ = −3.2, *p* < 0.01], whereas performance was similar in the saline conditions between the two tasks [*t*_(14)_ = 1.2, *p* = 0.2]. There was no significant effect of drug condition on response latency in the SSM task [*F*_(1, 15)_ = 1.0, *p* = 0.3; Figure 4B].

Overall, the results demonstrate that the dorsal hippocampus was critical for responding conditionally to the same object (i.e., jar) when visual scenes were used as critical information for decision making. Surprisingly (and somewhat contrary to the literature), the dorsal hippocampus was also required when the critical information for behavioral choice was a so-called elemental stimulus, although the performance deficits were more severe when visual scenes were used.

### Single sensory stimuli required the hippocampus in some rats, but not in others

**Figure 5:**
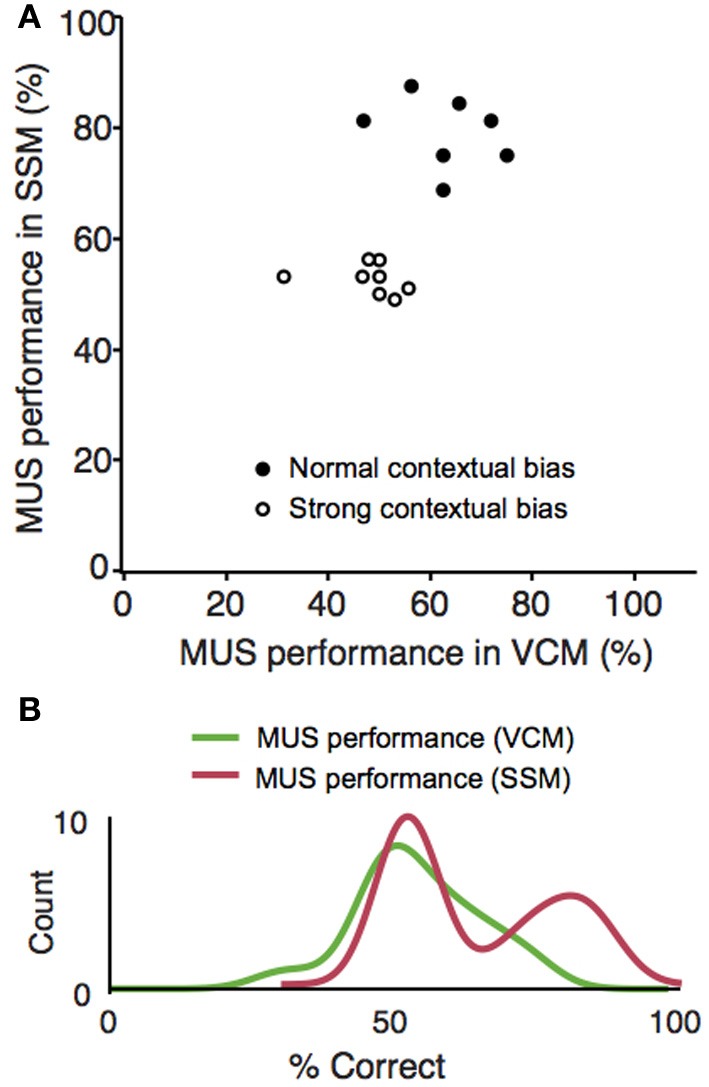
**Individual differences in responding to elemental cues in a hippocampal-dependent manner. (A)** Scatter plot for simultaneously showing the VCM and SSM task performances of the same animals after muscimol injections. The abscissa represents the performance (percent correct trials) in the VCM task and the ordinate represents the performance in the SSM task. Random jitters (<5) were added on the x and y coordinates of data points to make overlapping data points visible. A clear separation of the data points into two clusters can be seen for the MUS performance in the SSM task (clustering done by k-means clustering analysis). Rats showing performance deficits under MUS in the SSM task (“strong” contextual bias group) were marked with open circles and the rats with normal performance in the same conditions (“normal” contextual bias group) were marked with filled circles. **(B)** Data shown in **(A)** were plotted as separate distributions for the VCM and SSM tasks. The bimodal distribution is clearly visible (matching the separate clusters in the scatter plot) in the SSM task, but not in the VCM task.

Although rats were impaired in both the VCM and SSM tasks when muscimol was injected in the hippocampus, rats appeared to perform better in the SSM task than in the VCM task under muscimol in the hippocampus (Figure 4A). We suspected that this might be due to individual differences among rats. To examine this, we plotted performance of individual rats under the muscimol condition in the VCM task against performance under the muscimol condition in the SSM task (Figure 5A). A k-means clustering analysis run on the scatter data categorized data points into two separate clusters (Figures 5A,B). One cluster (filled circles in Figure 5A and the second red bump in Figure 5B) contained rats whose performance under hippocampal muscimol was close to chance (50%) in the VCM task but significantly above chance in the SSM task. These rats were considered as rats with “normal” contextual biases because they required the hippocampus for processing the visual scene but not for simple sensory stimuli. The other cluster contained rats whose performance under muscimol was close to chance in both the VCM task and the SSM task (open circles in Figure 5A; the first red bump in Figure 5B). These rats were considered as “strong” contextual rats because they required the hippocampus to process both visual scene and simple sensory stimuli. We found no differences in the cannula tip positions of the normal and strong contextual rats (Figure 3).

**Figure 6:**
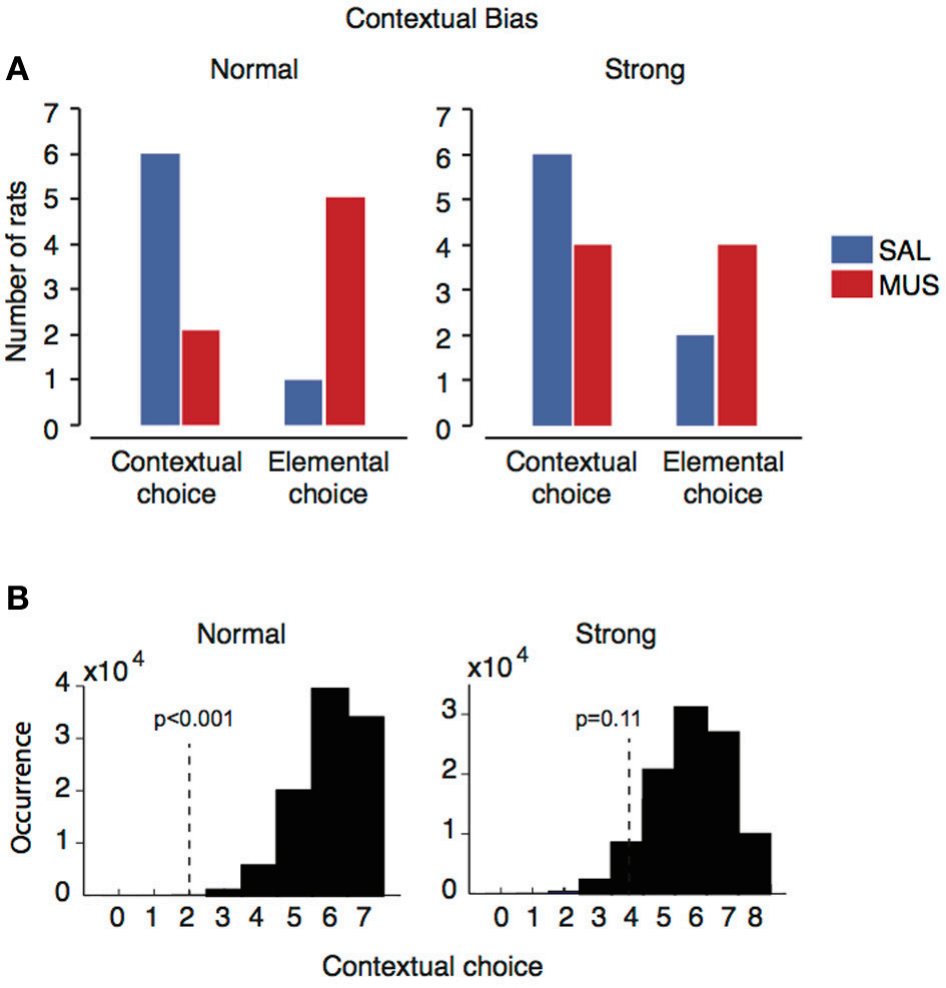
**Individual difference in contextual bias in the presence and absence of the hippocampus. (A)** The number of rats showing contextual or elemental responses when contextual and elemental cues with conflicting responses were simultaneously presented in probe trials. Only the responses from the first incongruent trials were used for preventing learning effects etc. In the normal contextual bias group, the majority of rats (*n* = 6) chose contextual responses when the hippocampus was functioning normally (SAL), but the same rats switched to elemental responses when the hippocampus was not available (MUS). In the strong contextual bias group, however, such a dramatic shift under MUS was not observed although the same contextual response preference was observed with SAL as in the normal contextual bias group. **(B)** The likelihood distribution for the normal contextual group (*left*) and the strong contextual group (*right*) under vehicle conditions, obtained by bootstrapping repetitive sampling procedures (*n* = 100,000). The location of the observed frequency of the contextual response under muscimol is shown as a vertical dotted line with the probability of observing the case in the SAL distribution.

To test if there were hierarchical relationships between different types of sensory stimuli, in our probe task, visual context and simple sensory stimuli were presented in conflict with each other in some trials (incongruent trials; Figure 2C). When considering only the first incongruent trial, most rats (12 out of 15; six out of seven in the normal context group and six out of eight in the strong context group) chose the response associated with the visual scene under vehicle conditions (Figure 6A). However, when the hippocampus was inactivated by muscimol, five out of seven rats in the normal contextual group chose the response associated with the simple sensory stimulus (elemental choice), whereas rats in the strong contextual group appeared to choose the response at random (Figure 6A). That is, four of the eight rats chose the response associated with the visual context (contextual choice) and the other half of rats chose the response associated with the simple sensory stimulus (elemental choice). However, presumably due to the small sample sizes, the numbers of rats that exhibited the responses associated with contextual and elemental responses (under muscimol injections) were not significantly different between the strong and normal context groups (*p* = 0.2, Fisher's exact test; *p* = 1.0 in the saline condition).

In order to overcome the small sample sizes, in each group (i.e., normal contextual group and strong contextual group), we calculated the probability that the rats with hippocampal inactivations would show contextual responses during the incongruent trial with the same likelihood of occurrence as in the saline conditions. A bootstrap resampling procedure was used for this. Specifically, in the normal contextual group (Figure 5A), six out of seven rats showed contextual responses in the incongruent trials (blue bars in “normal” in Figure 6A). By randomly sampling response preferences for 100,000 times (repetition allowed) out of the normal contextual group's response profile, we were able to obtain a probability distribution of the normal rat choosing the contextual response over the elemental response when the two types of responses were in conflict with each other in the incongruent trials (Figure 6B). In the normal contextual bias group, the likelihood of observing the muscimol condition-like response profile (i.e., 2:5 for contextual:elemental responses; red bars in “normal” in Figure 6A) in the saline-injected rats was less than 0.001 (one-tailed) according to the distribution obtained from the bootstrap procedures. The results support the interpretation that the rats with muscimol injected in the hippocampus showed an abnormal shift to the elemental response preference (from the contextual response preference). When the same bootstrapping method was applied to the strong contextual group, the likelihood of the saline-injected rats would show a similar response profile of the muscimol-injected condition (4:4 for contextual:elemental responses; red bars in “strong” in Figure 6A) was 0.11 (one-tailed; Figure 6B). This suggests that the rats in the strong contextual bias group did not behave differently between muscimol and saline conditions.

Regardless of the statistical sampling issues, the clear separation of rats into two clusters with respect to the hippocampal dependence of processing contextual and elemental cues (Figure 5A) strongly indicates that there is a large variability among rats in processing simple sensory cues in a hippocampal-dependent manner.

### Tactile cues served as strong elemental stimuli that were not dependent on the hippocampus

**Figure 7:**
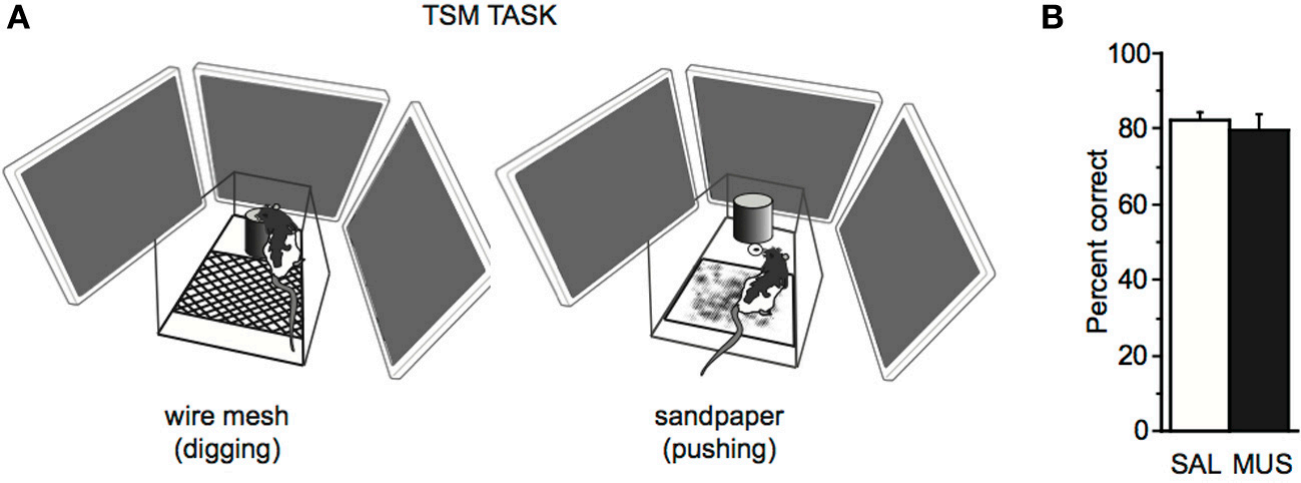
**TSM task. (A)** The TSM task was the same as the SSM task except that the tone and light stimuli used in the SSM task were replaced with a floor insert with wire mesh (*left*) or sandpaper (*right*) attached to its surface. To obtain a reward, the rat was required to dig in the sand in the jar (digging, *left*) or push the jar itself (pushing, *right*) in association with the tactile cue. **(B)** No significant performance difference was found in the TSM task between SAL and MUS conditions. Mean ± s.e.m.

Because rats belonging to the strong context group showed performance deficits in the SSM task, we suspected that those rats might have processed the simple sensory stimuli as background cues, as they did for the visual scene stimuli in the VCM task. To further examine this, we tested rats in Group 2 in a TSM task (Figure 7A) whereby the response was cued by a floor insert with either wire mesh or sandpaper texture. We reasoned that this type of cue had a lower possibility of being perceived as a background cue because the rat's paws touched the cue throughout the decision-making processes.

We found that rats performed well (showing approximately 80% performance level) regardless of saline or muscimol injection in the hippocampus in the TSM task. There was no significant effect of drug condition on performance in the TSM task [*F*_(1, 6)_ = 0.18, *p* = 0.7, One-Way repeated-measures ANOVA; Figure 7B], and the rats categorized into the normal or the strong context group were not different from each other in performance in the TSM task [*F*_(1, 5)_ = 0.2, *p* = 0.6, One-Way ANOVA]. The results suggest that rats do not require the hippocampus for associative memory for tactile stimuli.

## Discussion

In the current study, we compared the performance of rats in the hippocampal-dependent task with the performance in other tasks that required association of the same behaviors with cues that are traditionally considered to be elemental stimuli, i.e., simple tone, light, and tactile cues. We found that although the visual context cues were hippocampal and the tactile cues were non-hippocampal, there were individual differences among rats in the use of the hippocampus for associating simple tone and light stimuli with behavioral responses. In our study, all cues (i.e., visual context, light, tone, and tactile cues) were available up to the point where the rat made a choice response. Therefore, it is unlikely that the differential results stemmed from requiring a trace memory in some paradigms but not in others.

We previously showed that associating certain behavioral responses with visual stimuli in the background critically requires the dorsal hippocampus (Kim et al., 2012). However, the behavioral responses in that study required the rats to touch a response box (visual image) that appeared either on the left or right side of a monitor; therefore, one might think that the task was hippocampal dependent simply because a spatial behavior was required as response. In the current study, we trained rats to associate visual scenes with non-spatial behaviors, i.e., digging or pushing a sand-filled jar in a fixed location. Therefore, the hippocampal disruption of performance in the current study cannot be attributed to the requirement of a spatial behavior. The performance deficits similarly observed between the two versions (i.e., touching response images on different sides in the previous paradigm versus pushing-digging a sand-filled jar in the current study) strongly suggest that the hippocampus is necessary as long as the rat is required to make a response selection toward a common object by using the visual information in its background (Lee and Lee, 2013). The results also make it highly unlikely that the rats perceive a visual scene presented in the background LCD monitors as a simple object stimulus because simple object memory is not dependent on the hippocampus unless contextual and/or spatial variables are associated (Lee and Solivan, 2008; Winters et al., 2008; but see Broadbent et al., 2010). In our previous study (Kim et al., 2012), when the rat was required to treat the visual stimulus as an object by presenting different visual patterns in the side monitors and requiring the animal to go toward the monitor on which a rewarding visual pattern was displayed, hippocampal inactivations resulted in no performance deficits.

The individual differences reported in the current study for necessitating the dorsal hippocampus for associating simple sensory stimuli with different behavioral responses seem at odds with the relatively consistent reports of simple sensory stimuli not being dependent on the hippocampus in the fear-conditioning literature (Kim and Fanselow, 1992; Phillips and LeDoux, 1992, 1994). However, the hippocampus may well be involved in processing both contextual and non-contextual stimuli. For example, in a trace eyeblink conditioning task, changes in synaptic strength were noticed among the hippocampal subfields when an elemental stimulus (CS) was associated with an air puff (US), whereas synaptic connections were strengthened between the entorhinal cortex and the hippocampus when only a context (the environment in which the CS-US associations were taking place) was associated with the US in the absence of the CS (Carretero-Guillén et al., 2013). Furthermore, some prior studies report effects of dorsal hippocampal manipulations on fear conditioning to a tone stimulus (Maren et al., 1997; Bast et al., 2003). Bast et al. speculated that dorsal hippocampal manipulations might have disrupted mnemonic processes in the ventral hippocampus, which is connected to the amygdala, an area critical for fear conditioning (Bast et al., 2003). Maren et al. suggested that the tone stimulus may have been processed as a configural stimulus and processed via the hippocampus (Maren et al., 1997). However, neither of these studies examined individual differences among rats (Maren et al., 1997; Bast et al., 2003) as in the current study. As our findings suggest that rats may be categorized as either weakly or strongly contextual, a parsimonious explanation of previous results could be that there were more strongly contextual rats than weakly contextual rats in the subject population (Maren et al., 1997; Bast et al., 2003). It is also possible that the flight-and-fight nature of the fear-conditioning experiment makes rats more biased to become less contextual, enabling the defensive behavior to be expressed flexibly and rapidly based on other individual cues in the absence of the hippocampus.

In our SSM task, tone stimulus was provided through a pair of computer speakers located underneath the platform and the light stimulus was provided through a light bulb attached to one of the sidewalls of the platform. In comparison to the contextual stimulus, which was provided as a surrounding visual context through the LCD panels, tone and light cues were more focal and less incorporated into the background of the environment. However, it is important to note that all sensory cues were available persistently from the moment the start box door was opened. Therefore, some rats might have perceived the visual and auditory conditions around the platform differently in light and no-light (i.e., tone) trials. That is, the stimulus might have been interpreted as being present in the background by some rats but in the foreground by others. For example, some rats might have considered the light as a single stimulus, whereas others may have focused on a local environment illuminated by the light. These differences in the perception of sensory stimuli might have resulted in differences among individual rats in remembering the cued behaviors in a hippocampal-dependent manner. By contrast, in the TSM task, as rats continuously touched the local tactile cue (textured floor insert) throughout the decision-making process, there might be relatively little room for the interpretation of this tactile cue as being part of the background.

A dominant theory posits that there are spatial and non-spatial information processing streams in the cortical areas outside the hippocampus, and the hippocampus integrates the spatial and non-spatial streams into a unified event representation (Burwell, 2000; Hargreaves et al., 2005; Knierim et al., 2006; Manns and Eichenbaum, 2006). However, the potential inadequacy of this simple dichotomy was recently pointed out (Lee and Lee, 2013; Knierim et al., 2014). Our current findings also suggest that the simple and dichotomous categorization of a stimulus (e.g., spatial or non-spatial; contextual or elemental) may not be relevant in some cases, and that the categorization might be further affected by individual differences as well as task demands. This might explain the inconsistencies in the results of early studies of hippocampal-dependent learning of elemental and configural stimuli (Sutherland and Rudy, 1989; Gallagher and Holland, 1992; Davidson et al., 1993) and fear-conditioning studies (Kim and Fanselow, 1992; Phillips and LeDoux, 1992, 1994; Maren et al., 1997; Bast et al., 2003). Our study thus provides cautionary evidence that the elemental versus contextual, or hippocampal versus non-hippocampal, characterization of a stimulus should be made on the basis of behavioral testing in a given paradigm, but not entirely dependent on the literature only.

## Conflict of interest statement

The authors declare that the research was conducted in the absence of any commercial or financial relationships that could be construed as a potential conflict of interest.
